# De novo assembly and analysis of the *Artemisia argyi* transcriptome and identification of genes involved in terpenoid biosynthesis

**DOI:** 10.1038/s41598-018-24201-9

**Published:** 2018-04-11

**Authors:** Miaomiao Liu, Jinhang Zhu, Shengbing Wu, Chenkai Wang, Xingyi Guo, Jiawen Wu, Meiqi Zhou

**Affiliations:** 1 Graduate School, Anhui University of Chinese Medicine, Hefei, 230038 China; 2 Department of Physiology, School of Basic Medical Sciences, Anhui Medical University, Hefei, 230032 China; 3 Key Laboratory of Xin’an Medicine, Ministry of Education, Anhui University of Chinese Medicine, Hefei, 230038 China; 4 Institute of Acu-moxibustion and Meridian-collaterals, Anhui University of Chinese Medicine, Hefei, 230038 China; 5 Division of Epidemiology, Department of Medicine, Vanderbilt University School of Medicine, Nashville, TN 37232 USA; 6 Synergetic Innovation Center of Anhui Authentic Chinese Medicine Quality Improvement, Hefei, 230038 China

## Abstract

*Artemisia argyi* Lev. et Vant. (*A. argyi*) is widely utilized for moxibustion in Chinese medicine, and the mechanism underlying terpenoid biosynthesis in its leaves is suggested to play an important role in its medicinal use. However, the *A. argyi* transcriptome has not been sequenced. Herein, we performed RNA sequencing for *A. argyi* leaf, root and stem tissues to identify as many as possible of the transcribed genes. In total, 99,807 unigenes were assembled by analysing the expression profiles generated from the three tissue types, and 67,446 of those unigenes were annotated in public databases. We further performed differential gene expression analysis to compare leaf tissue with the other two tissue types and identified numerous genes that were specifically expressed or up-regulated in leaf tissue. Specifically, we identified multiple genes encoding significant enzymes or transcription factors related to terpenoid synthesis. This study serves as a valuable resource for transcriptome information, as many transcribed genes related to terpenoid biosynthesis were identified in the *A. argyi* transcriptome, providing a functional genomic basis for additional studies on molecular mechanisms underlying the medicinal use of *A. argyi*.

## Introduction

*Artemisia argyi* (*A. argyi*) Lev. et Vant., a perennial herb belonging to the genus *Artemisia* and the family Asteraceae, is widely distributed in China^1^. *A. argyi* leaves have long been used extensively for a form of traditional Chinese medicine (TCM) known as moxibustion^2^. Previous phytochemical studies on *A. argyi* leaves have revealed the presence of large amounts of volatile oils that have antihistamine^3^, antifungal and antiviral effects as well as the abilities to eliminate phlegm and relieve asthma and coughing^4,5^. In addition, dried and ground *A. argyi* leaves are the original material for moxa floss, which is used for moxibustion as a TCM therapeutic to cure dysmenorrhea^6^, diarrhoea^7^ and fatigue^8^, and *A. argyi* leaf volatile oils play a significant therapeutic role in moxibustion.

The main components of *A. argyi* leaf volatile oils are monoterpenes and sesquiterpenes. The mevalonate (MVA) and 2-C-methyl-D-erythritol 4-phosphate (MEP) pathways are, respectively, responsible for synthesizing isopentenyl pyrophosphate (IPP) and dimethylallyl pyrophosphate (DMAPP). IPP and DMAPP are the precursor substances for terpenoid^9^, and they can be converted into each other by IPP isomerase (IPPI). DMAPP is catalysed to form geranyl diphosphate (GPP) by GPP synthase (GPPS), and IPP is converted into farnesyl diphosphate (FPP) by FPP synthase (FPPS)^10^. Then, through the actions of monoterpene synthase and sesquiterpene synthase (Sesqui-TPS), monoterpenes and sesquiterpenes are produced from the precursors GPP and FPP, respectively^11^. Moreover, transcription factors (TFs) that reportedly regulate terpenoid synthesis are mainly concentrated in the APETELA2/ethylene-responsive binding protein (AP2/EREBP)^12^, WRKY^13^, basic leucine zipper (bZIP)^14^, and basic helix-loop-helix (bHLH)^15^ families.

RNA sequencing (RNA-seq) is the best method for screening functional genes and evaluating the expression of genes without a reference genome^16,17^. Currently, RNA-seq has been performed on dozens of medicinal plants, including *Artemisia annua*^18^, *Glycyrrhiza uralensis*^19^, *Lonicera japonica*^20^, *Carthamus tinctorius*^21^, *Lilium regale*^22^ and *Eugenia uniflora*^23^, providing an effective way to identify new gene functions in specific metabolic pathways^24^. As no transcriptomic data are currently available for *A. argyi*, we performed RNA-seq via the Illumina HiSeq. 4000 sequencing platform to assemble the *A. argyi* transcriptome. This process yielded a total of 99,807 unigenes, most of which were annotated in public databases, and numerous genes related to terpenoid biosynthesis were identified. Our transcriptomic data provide a valuable resource for future studies on the molecular mechanisms of terpenoid biosynthesis and may increase the yield of volatile oil from *A. argyi*.

## Results

### RNA-seq and de novo transcriptome assembly

Illumina high-throughput sequencing of the *A. argyi* transcriptome generated approximately 74 billion clean reads from each tissue (Supplementary Table S1). After the clean reads were sequentially assembled, clusters were made, and redundant clusters were removed with the Trinity and TGI clustering tool (TGICL), a total of 99,807 unigenes were obtained, with a median length of 929 bp (Supplementary Table S2). The N50 length and average GC% were 1456 bp and 40.79%, respectively. Of these unigenes, 56.6% (56,480) were longer than 500 bp, and 32.2% (32,112) were longer than 1000 bp (Supplementary Fig. S1A). Compared to the *A. annua* transcriptome as assembled on the Illumina HiSeq. 2500 platform^25^, this study presents more unigenes, a longer N50 length, and a longer mean length, suggesting that these data are highly reliable. In addition, the identities of a total of 59,944 coding sequences (CDSs, average length of 775 bp), 63.5% (38,066) of which were longer than 300 bp (Supplementary Fig. S1B), were predicted using BLAST.

### Unigene functional annotations

Functional annotation analysis showed that of the 67,446 unigenes, 60.67%, 44.04%, 25.69%, 20.13%, 44.84%, 42.46% and 45.16% acquired significant hits in the Nr (Non-redundant), Nt (Nucleotide), COG (Cluster of Orthologous Groups of Proteins), GO (Gene Ontology), KEGG (Kyoto Encyclopedia of Genes and Genomes), Swiss-Prot and InterPro databases, respectively (Table 1). As artemisia does not belong to a family containing a model organism, it is logical that many unigenes in *A. argyi* were not annotated with GO terms^26^. A total of 19,256 (29%) unigenes were co-annotated in the five databases (Supplementary Fig. S2A). Based on the Nr annotation, distributions of homologous *A. argyi* species were calculated, and 51.89% of the unigenes had the highest homology with *Cynara cardunculus* (Compositae), followed by *Vitis vinifera* (Vitaceae), and *Sesamum indicum* (Pedaliaceae) (Supplementary Fig. S2B). The functions of the unigenes were predicted via GO enrichment, using terms divided into three classes, molecular function, cellular component and biological process, comprising 54 functional categories (Supplementary Fig. S3). In the biological process class, “metabolic process” was among the most common categories. The term “metabolic process” was also among the most common types of biological process according to GO analysis based on pyrosequencing of expressed sequence tags (ESTs) of the *A. annua* glandular trichome^18^, indicating the importance of metabolic activities in both *A. argyi* tissues and *A. annua* glandular trichomes.

**Table 1:** Annotation of unigenes against seven different databases.

Annotated database	Number of annotated unigenes	Annotated unigene ratio (%)
Nr	60,554	60.67
Nt	43,951	44.04
COG	25,638	25.69
GO	20,091	20.13
KEGG	44,750	44.84
Swiss-Prot	42,374	42.46
InterPro	45,068	45.16
All	67,446	67.50

### Identification of genes involved in terpenoid backbone biosynthesis by KEGG analysis

**Figure 1:**
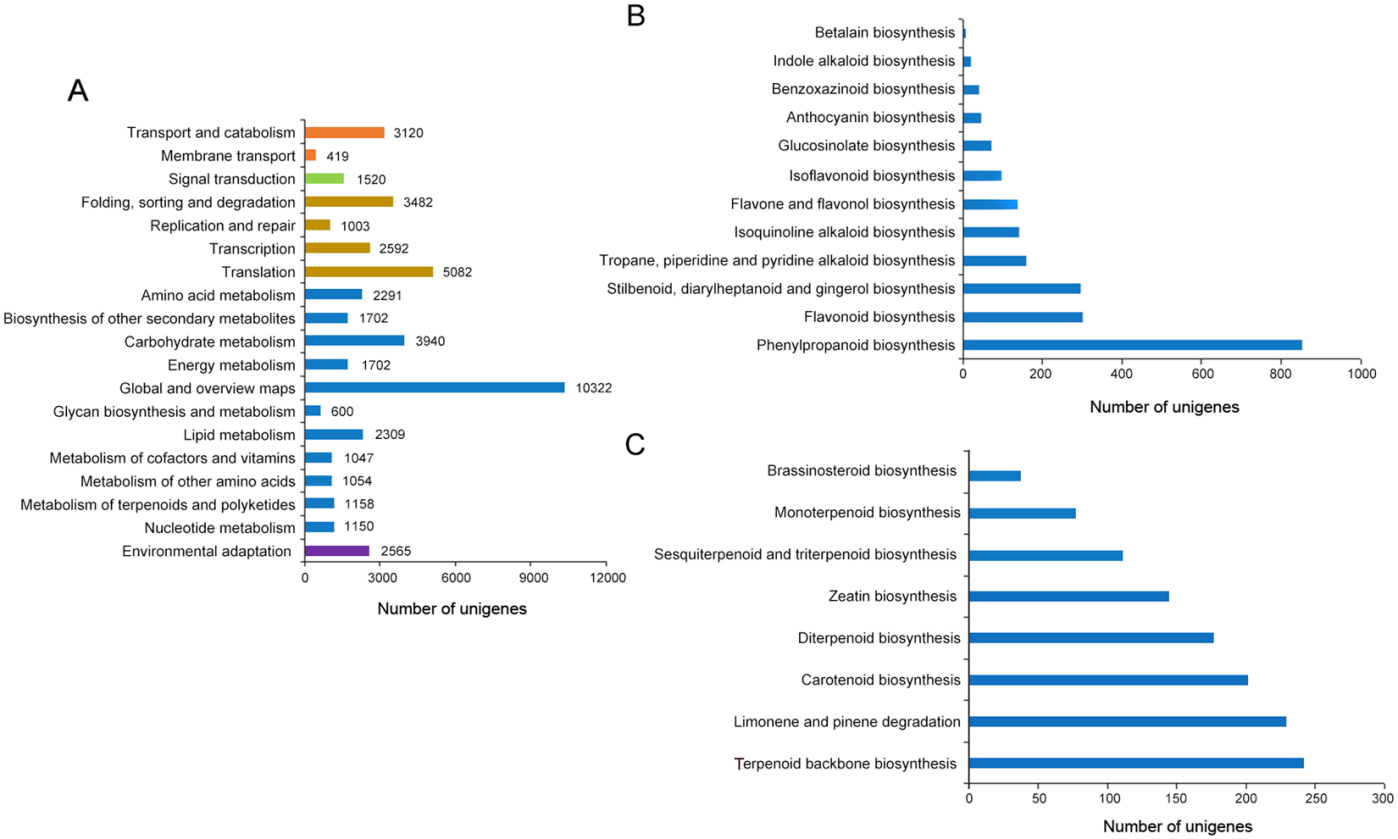
KEGG annotation of *A. argyi* unigenes. (**A**) KEGG functional classifications of the assembled unigenes. The unigenes were divided into five primary categories: genetic information processing (orange), cellular processes (green), organismal systems (brown), metabolism (blue), and environmental information processing (purple). (**B**) Classifications based on biosynthesis of other secondary metabolites. (**C**) Classifications based on metabolism of terpenoids and polyketides.

**Figure 2:**
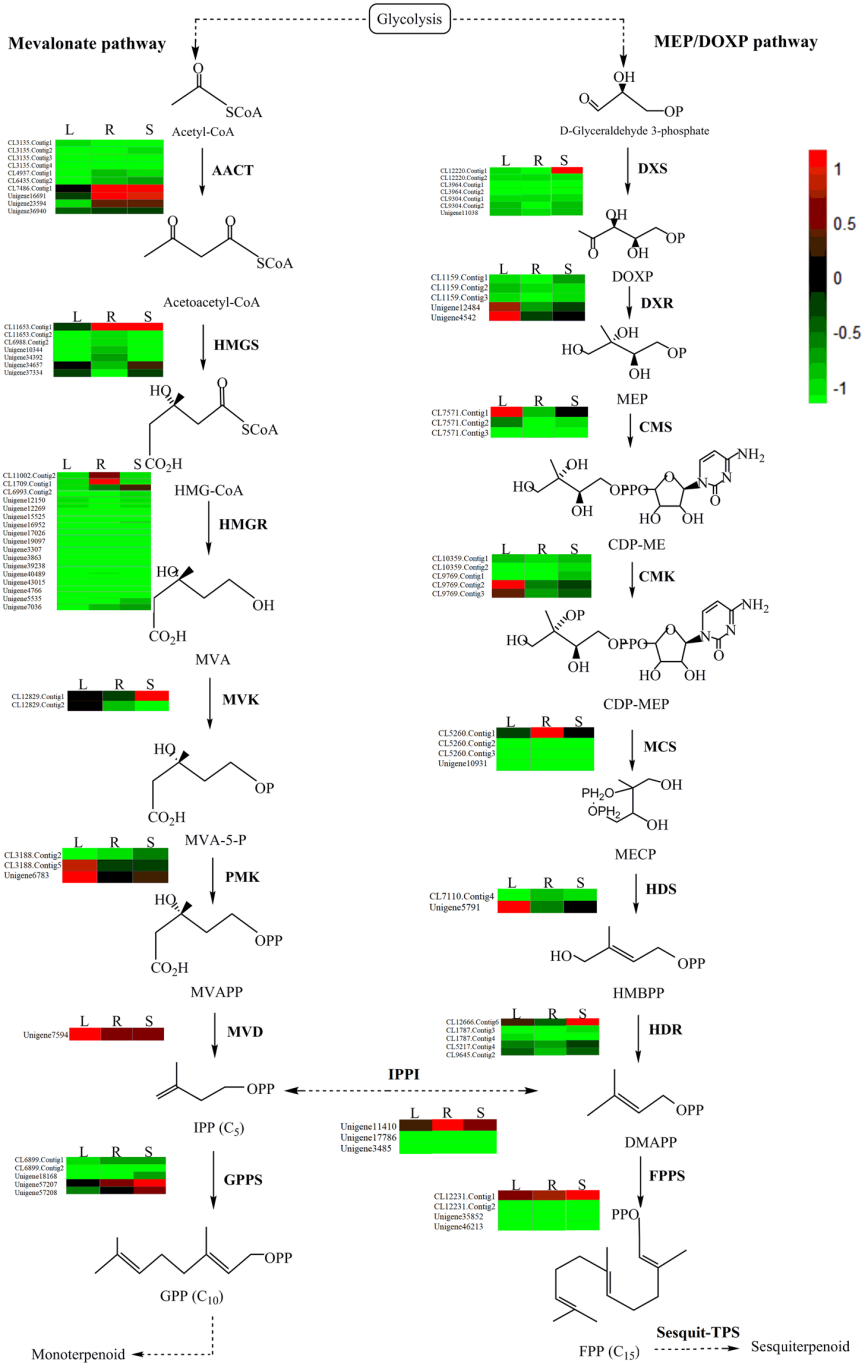
The monoterpene and sesquiterpene biosynthesis pathway in *A. argyi*. The expression levels of unigenes encoding enzymes from each step are shown. The columns are L, R, S, corresponding to leaf, root and stem, respectively, and the rows correspond to unigenes. Red and green represent high and low expression levels, respectively.

To discover the most significant biological pathways, 44,750 unigenes were annotated in the KEGG database and classified into five classes, cellular process, genetic information processing, metabolism, organismal systems and environmental information processing, comprising 19 subcategories (135 pathways) (Fig. 1A). A total of 12 pathways were involved in the biosynthesis of other secondary metabolites, among which the most genes were enriched in the phenylpropanoid biosynthesis pathway (Fig. 1B). The “metabolism of terpenoids and polyketides” subcategory contained 8 pathways, and the largest number of unigenes (241) were mapped to terpenoid backbone biosynthesis (Fig. 1C). Among these 241 unigenes, 114 were identified as encoding 16 key enzymes that control terpenoid biosynthesis, including acetyl-CoA acetyltransferase (AACT), hydroxymethylglutaryl-CoA synthase (HMGS), hydroxymethylglutaryl-CoA reductase (HMGR), mevalonate kinase (MK), phosphomevalonate kinase (PMK), mevalonate diphosphate decarboxylase (MVD), 1-deoxy-D-xylulose-5-phosphate synthase (DXS), 1-deoxy-D-xylulose-5-phosphate reductoisomerase (DXR), 2-C-methyl-D-erythritol 4-phosphate cytidylyltransferase (CMS), 4-diphosphocytidyl-2-C-methyl-D-erythritol kinase (CMK), 2-C-methyl-D-erythritol 2,4-cyclodiphosphate synthase (MCS), 4-hydroxy-3-methylbut-2-enyl diphosphate synthase (HDS), 4-hydroxy-3-methylbut-2-enyl diphosphate reductase (HDR), IPPI, FPPS and GPPS (Table 2). These unigenes are mainly distributed in the MEP (62 unigenes, 6 enzymes) and MVP (43 unigenes, 8 enzymes) pathways upstream of terpenoid synthesis, while a few genes (9 unigenes, 2 enzymes) are distributed downstream (Fig. 2).

**Table 2:** Unigenes involved in the terpenoid biosynthesis pathway in *A. argyi*.

Enzyme name	EC number	Unigene number	No. in leaves	No. in roots	No. in stems
AACT	2.3.1.9	15	8	10	8
HMGS	2.3.3.10	12	3	8	3
HMGR	1.1.1.34	27	20	24	19
MK	2.7.1.36	3	2	2	2
PMK	2.7.4.2	4	4	4	4
MVD	4.1.1.33	1	1	1	1
DXS	2.2.1.7	9	9	9	7
DXR	1.1.1.267	5	5	5	5
CMS	2.7.7.60	4	3	3	3
CMK	2.7.1.148	5	3	4	5
MCS	4.6.1.12	4	4	4	4
HDS	1.17.7.1	3	1	2	3
HDR	1.17.7.2	9	4	6	6
IPPI	5.3.3.2	4	3	3	2
FPPS	2.5.1.10	4	1	4	3
GPPS	2.5.1.1	5	4	4	5

### Overview of unigene expression

**Figure 3:**
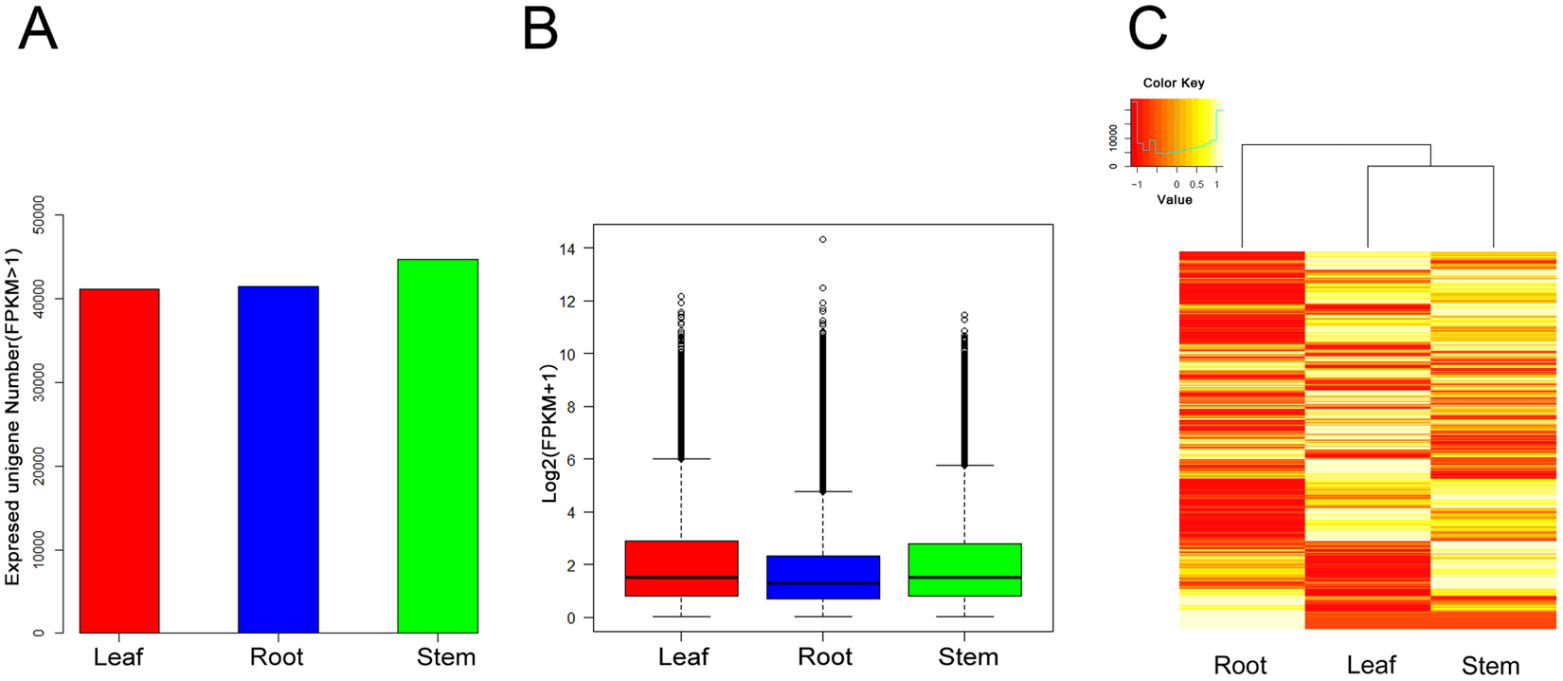
Overview of expression profiles in the three *A. argyi* tissues. (**A**) Expressed unigene (FPKM > 1) number distributions in the three tissues. (**B**) Boxplot of unigenes expressed in the three tissues. The samples are represented on the x-axis, and the log2 (FPKM + 1) values are represented on the y-axis. (**C**) Heatmap of unigenes co-expressed in the three tissues. The intensity of the colour scheme is scaled to the log2 (FPKM + 1) expression values that are Z-score-standardized per transcript in the samples, and yellow and red represent high and low expression levels, respectively.

In each sample, all of the expressed unigenes (fragments per kilobase of transcripts per million fragments mapped (FPKM) > 1)^27^ were determined, and 41,139, 41,516 and 44,750 unigenes were expressed in leaves, roots and stems, respectively (Fig. 3A). The overall expression levels were the highest for leaf transcripts, followed by the stem and root transcripts (Fig. 3B). Transcripts expressed at low levels in the three tissues were filtered with a geometric mean (FPKM + 1) < 3 as the threshold^27^, generating 43,023 unigenes in these tissues. Hierarchical clustering of the three tissues with these 43,023 unigenes showed that leaves and stems clustered more tightly, demonstrating that the overall expression levels of transcripts in these two tissues were more closely related (Fig. 3C).

### Identification of genes with leaf-specific expression and differentially expressed genes

**Figure 4:**
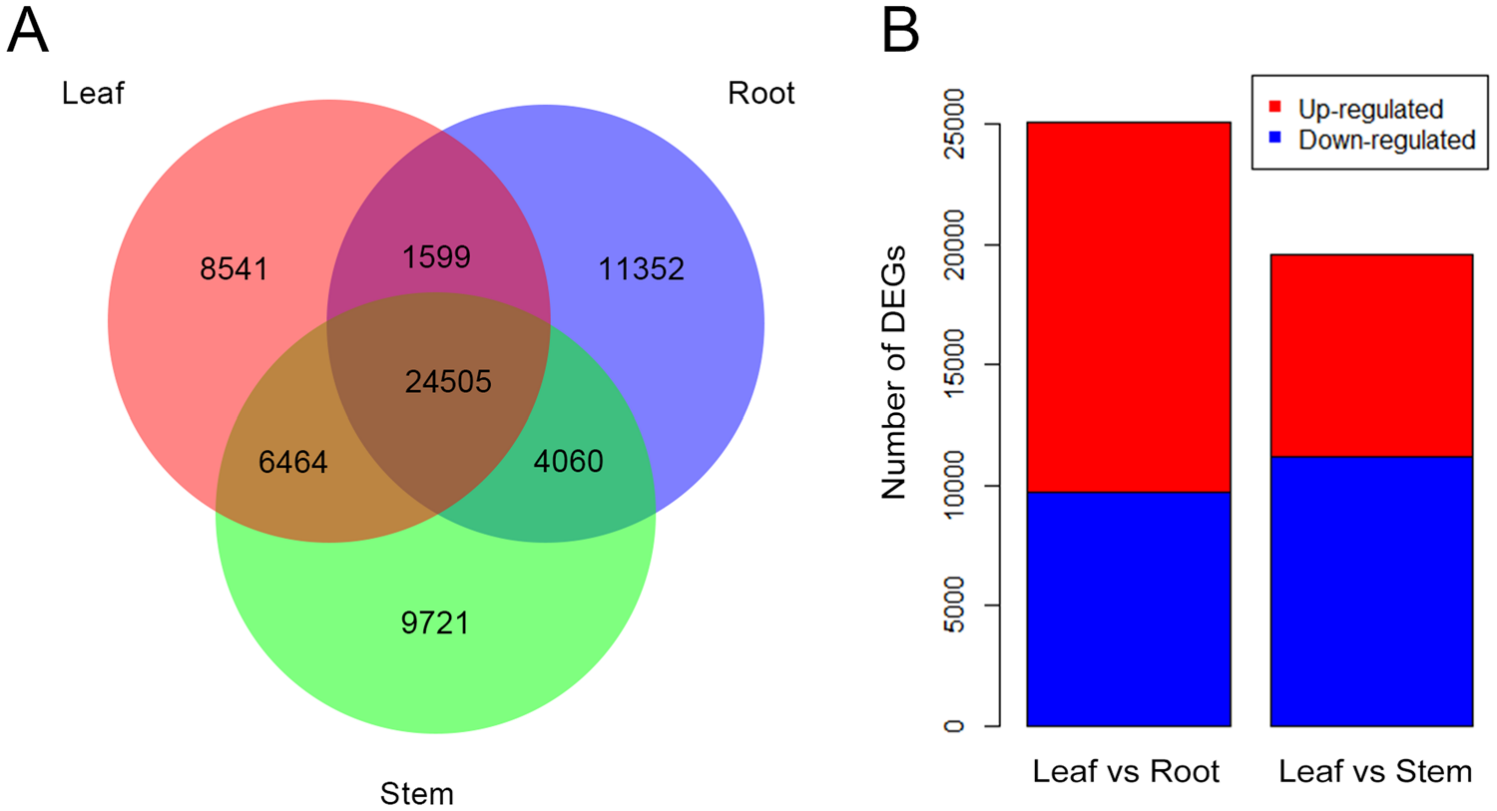
Unigenes expressed in different *A. argyi* tissues. (**A**) Venn diagram of unigenes expressed in different tissues. (**B**) Statistic of DEGs in the three *A. argyi* tissues. The numbers of up-regulated and down-regulated unigenes between the two indicated samples are summarized. DEGs with higher expression levels in leaves than in roots or stems were defined as “up-regulated”, while those with lower expression levels in leaves were defined as “down-regulated”.

A total of 24,505 shared unigenes were identified in all three tissues, and 8,541 were uniquely expressed in leaves (Fig. 4A). Among the shared unigenes expressed in all tissues, 603 showed leaf-specific up-regulation with fold changes (FCs) > 8, and these genes were further evaluated using GOSlim functional analysis. Based on sequence homology, these 603 unigenes were assigned to one or more ontologies, including 260 for cellular component, 328 for biological process, and 389 for molecular function (Supplementary Table S3). In the biological processes category, several genes were enriched for the term “secondary metabolic process”, indicating important metabolic activities in leaves.

The total differentially expressed genes (DEGs) were detected among the samples by using unigene expression analysis (Fig. 4B). Comparison of the leaves and roots revealed 25,049 DEGs, of which 15,376 were up-regulated (higher expression in leaves) and 9,673 were down-regulated (lower expression in leaves). Comparison of the leaves and stems revealed 19,588 DEGs, of which 8,409 were up-regulated in the leaves and 11,179 were down-regulated. To further connect the biological functions of the DEGs, all DEGs were mapped to the KEGG database and compared to the entire *A. argyi* transcriptome. Indeed, 134 pathways, primarily related to metabolism, biosynthesis of secondary metabolites and plant-pathogen interactions, were enriched in DEGs (Supplementary Table S4). The “metabolism of terpenoids and polyketides” subcategory was particularly enriched in DEGs. Overall, 251 genes were up-regulated in leaves compared to roots, while 148 genes were up-regulated in leaves compared to stems (Table 3).

**Table 3:** The terpenoid and polyketide metabolic pathway and the numbers of related DEGs in leaves compared with the other two tissues.

Terpenoid and polyketide metabolic pathway	Pathway ID	Number of up-regulated genes	
		Leaf vs Root	Leaf vs Stem
Terpenoid backbone biosynthesis	Ko00900	51	17
Limonene and pinene degradation	Ko00903	54	47
Carotenoid biosynthesis	Ko00906	70	35
Diterpenoid biosynthesis	Ko00904	25	9
Zeatin biosynthesis	Ko00908	17	10
Sesquiterpenoid and triterpenoid biosynthesis	Ko00909	16	16
Monoterpenoid biosynthesis	Ko00902	1	11
Brassinosteroid biosynthesis	Ko00905	17	3

### Identification of transcription factors involved in terpenoid biosynthesis

**Figure 5:**
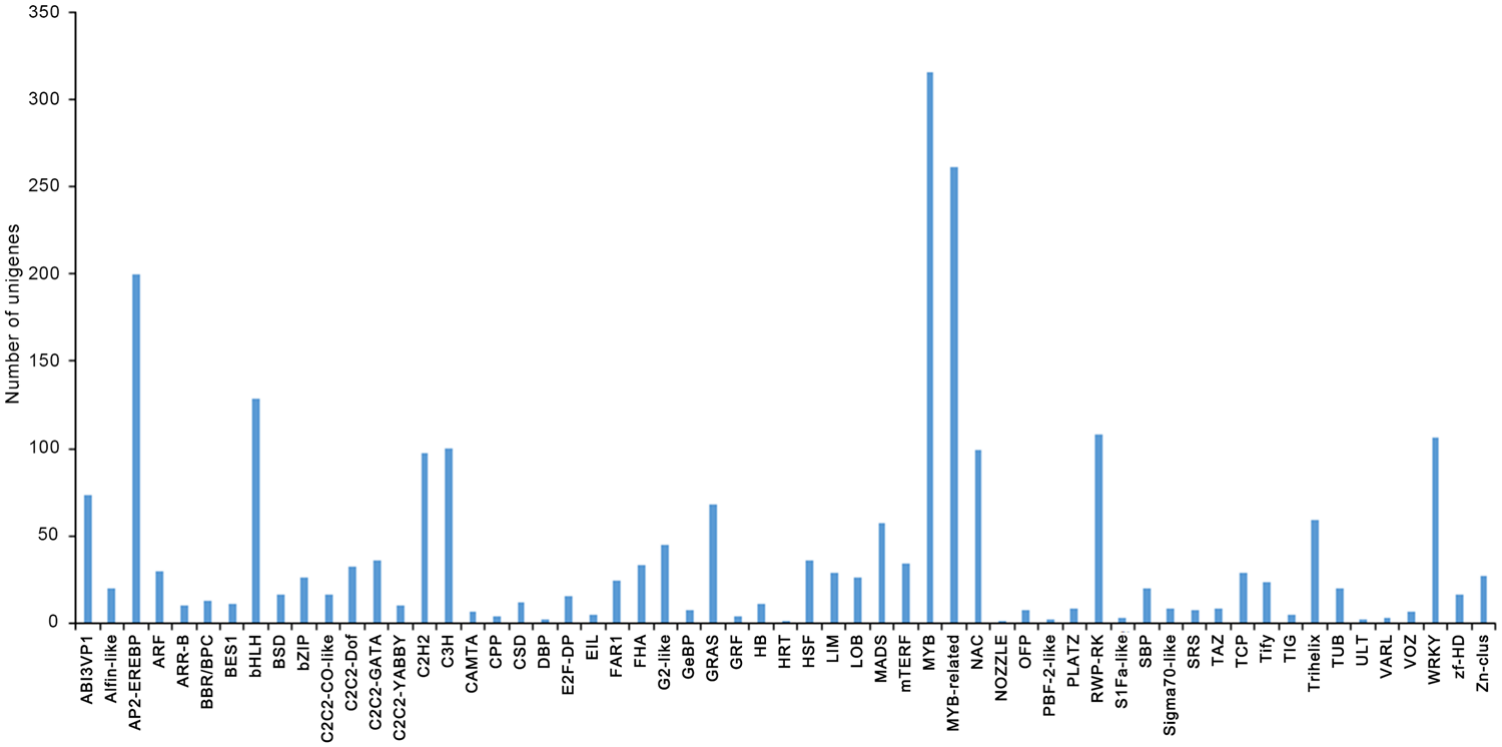
TF family classification of *A. argyi* unigenes.

In plants, TFs participate in a wide variety of biological processes and play major roles in regulating gene expression at the transcriptional level to control secondary metabolite flux. A total of 2,056 unigenes encoding TFs were identified and classified into 59 different TF families (Fig. 5). Among these TFs, 200, 129, 106 and 26 unigenes were annotated to the AP2-EREBP, bHLH, WRKY and bZIP families, respectively.

### Molecular cloning of candidate genes for terpenoid biosynthesis

Seven full-length open reading frames (ORFs) were cloned: ArHMGR1 (1758 bp), ArHMGR2 (1743 bp), ArMVD (1266 bp), ArDXS (2187 bp), ArDXR (1419 bp), ArHDS (2223 bp) and ArHDR (1365 bp) (Supplementary Fig. S4). Polymerase chain reaction (PCR) products were retrieved by gel extraction and ligated into the vector pMD19-T. The recombinant vectors were then transformed into DH5α *E. coli* competent cells for amplification, and these recombinant plasmids were verified by Sanger sequencing. The seven nucleotide sequences have been deposited in GenBank under accession numbers MG780995-MG781001.

## Discussion

To extend the possible applications of *A. argyi* in TCM, three different tissues were utilized for library construction and sequencing, and approximately 74 billion clean reads were generated from each tissue. In total, 99,807 and 67,446 unigenes were assembled and annotated, respectively, among which 19,256 were co-annotated in the databases. However, 33% of the unigenes remain unannotated, probably because more unigenes were generated with the sequencing depth of 10 G and because the publicly available plant transcriptome and genome data are insufficient. These predicted CDSs, accounting for 60.0% of the total unigenes, provide information for studying crucial genes, including genes encoding lectins^28^ and ribosome-inactivating proteins^29^, which are potential anticancer drugs.

The best hit for each unigene queried against the Nr database was used to assign functional GO annotations in terms of the categories cellular component, biological process and molecular function. The large number of diverse GO terms assigned to the unigenes highlights the diversity of genes likely represented in the *A. argyi* leaf, root and stem transcriptomes. Upon mapping these unigenes to the KEGG database, numerous unigenes involved in terpenoid biosynthesis were identified. In addition, we examined the expression levels of unigenes encoding enzymes in the MVP and MEP pathways based on FPKM values (Fig. 2). The unigenes encoding PMK, MVD, DXR, CMS, CMK and HDS were highly differentially expressed in leaves, suggesting that these steps may be rate-limiting in IPP and DMAPP formation, which occur upstream of terpenoid synthesis. Characterization of these unigenes will further improve our understanding of the molecular mechanisms underlying terpenoid biosynthesis.

The overall transcript expression level was higher in the leaves than in the roots and stems. According to the DEG annotation, hundreds of genes that were up-regulated in leaves were associated with the metabolism of terpenoids and polyketides. These up-regulated genes may be helpful for analysing terpenoid metabolites in *A. argyi*. In addition, the substantial numbers of genes showing leaf-specific expression and associations with secondary metabolic processes revealed the importance of metabolic activities in leaves. These genes showing leaf-specific expression or up-regulation might provide the transcriptomic support required to provide *A. argyi* leaves with their medicinal value.

In this work, 461 candidate TFs were assigned to the AP2-EREBP, bHLH, WRKY and bZIP families, and these TFs might play roles in regulating terpenoid biosynthesis. The bHLH transcription factor gene AabHLH1 in *A. annua* has been proven to effectively regulate the biosynthesis of the terpenoid artemisinin^30^. The use of genetic engineering methods to control TFs has substantial potential value and broad application prospects in studies on the regulation of terpenoid biosynthesis in *A. argyi*.

In this study, seven gene sequences, namely, those encoding ArHMGR1, ArHMGR2, ArMVD, ArDXS, ArDXR, ArHDS and ArHDR, were retrieved from the transcriptomic data and successfully cloned by PCR. These gene sequences were consistent with those identified from the *A. argyi* transcriptome, thus confirming the reliability of our transcriptional data. In addition, the expression level of HMGR, which was up-regulated in leaves, can reportedly increase the synthesis of artemisinin^31^, and the content of ginkgolide, another terpenoid, in transgenic *Ginkgo biloba* overexpressing HDR was significantly increased compared with that in the nontransgenic control line^32^. Therefore, our findings may help improve future studies on increasing the yield of terpenoids via gene regulation and the production of transgenic plants.

In summary, our study is the first exploration of the *A. argyi* transcriptome. We generated high-quality RNA-seq data from leaf, root and stem tissues of *A. argyi*. Using de novo transcriptome assembly, we assembled and annotated 99,807 and 67,446 unigenes, respectively. We analysed most of the unigenes encoding key enzymes involved in the terpenoid biosynthesis pathway and identified several TFs related to terpenoid synthesis. Our findings may help improve future studies on the molecular mechanisms of terpenoid biosynthesis and on increasing the yield of terpenoids via gene regulation and genetic engineering. Our transcriptomic dataset will also accelerate studies on *A. argyi* functional genomics.

## Materials and Methods

### Plant material and RNA extraction

Whole *A. argyi* plants (identified by Professor Qingshan Yang, Anhui University of Chinese Medicine) were harvested from the Anhui University of Chinese Medicine herb garden, cleaned with ultrapure water, dried on filter paper, and immediately soaked in liquid nitrogen after separation of the leaves, stems and roots. The leaves, roots and stems selected from five replicates were pooled together. Total RNAs from the plants were isolated with an RNA Plant Kit (Aidlab Biotech, Beijing, China) based on the manufacturer’s instructions. RNA quality was verified using an Agilent 2100 Bioanalyzer (Agilent Technologies, Palo Alto, CA, USA), and the average RNA Integrity Number (RIN) was 8.63.

### cDNA library construction and RNA sequencing

Total RNAs were treated with DNase I to eliminate DNA residues and then mixed with oligo (dT)-cellulose to purify the mRNAs. The purified mRNAs were fragmented, and first-strand cDNAs were synthesized using these mRNA fragments as templates. After the second-strand cDNAs were synthesized, the double-stranded cDNAs were randomly fragmented. Short cDNA fragments were recovered and repaired, and a single nucleotide (adenine) was added to the 3′ ends. The cDNA fragments were then joined to adapters, and the appropriate fragments were selected and used for PCR amplification. Each sample library was quantified and evaluated for quality on an Agilent 2100 Bioanalyzer and an ABI StepOnePlus Real-Time PCR System (ABI, New York, NY, USA), respectively. Ultimately, the one library per tissue was sequenced on the Illumina HiSeq 4000 platform (Beijing Genomics Institute, Wuhan, China). After sequencing, raw data were received, and low-quality reads and adapters were filtered to generate clean data.

### De novo transcriptome assembly

De novo transcriptome assembly was implemented using Trinity (version 2.06), which successively combines Inchworm, Chrysalis and Butterfly, to assemble clean reads^33^ with the following parameters: min contig length of 200 and min kmer coverage of 4. Ultimately, full-length transcripts for alternatively spliced isoforms were generated by splicing transcripts corresponding to paralogous genes. All such sequences were known as transcripts. Transcript analysis was performed to cluster and remove redundancies with TGICL (version 2.06, parameters: -l 30 -v 35) to acquire non-redundant sequences, termed unigenes^34^. All unigenes were segmented into two categories: clusters (prefixed with CL) and singletons (prefixed with unigene).

### Unigene expression analysis and functional annotation

After transcriptome assembly, clean data were mapped to unigenes with Bowtie2 (version 2.2.5, parameters:–phred64–sensitive–dpad 0–gbar 99999999–mp 1,1–np 1–score-min L,0,-0.1 -I 1 -X 1000–no-mixed–no-discordant -p 1 -k 200)^35^. Considering the FPKM values, the unigene expression level of each sample was computed using RSEM (version 1.2.12) with default settings^36^. When raw FPKM values were log2-transformed and used for downstream data analysis, a value of 1 was added to each raw FPKM value to avoid the emergence of log2(0)^37^.

To acquire unigene functional annotations, unigenes were aligned to protein databases, including Nt (ftp://ftp.ncbi.nlm.nil.gov/blast/db), Nr (ftp://ftp.ncbi.nlm.nil.gov/blast/db), COG (http://www.ncbi.nlm.nih.gov/COG), KEGG (http://www.genome.jp/kegg) and Swiss-Prot (http://ftp.ebi.ac.uk/pub/databases/swissport), using BLAST (version 2.2.23, E-value ≤ 1e-5)^38^. In addition, Blast2GO (version 2.5.0, default parameters)^39^ was used to generate GO annotations (http://www.geneontology.org) with Nr annotations, and InterPro annotations (http://www.ebi.ac.uk/interpro) were obtained using InterProScan5 software (version 5.11–51.0, default parameters)^40^. For the functional annotation analysis, unigenes that best mapped to functional databases in the priority order Nr, Swiss-Prot, KEGG, COG were selected by BLAST, defined as CDSs, and identified from the 5′ to 3′ ends.

### Identification of differentially expressed genes

For comparing unigene expression levels in two tissues, such as leaf vs root tissue and leaf vs stem tissue, unigenes with FCs ≥ 2.00 and false discovery rate values ≤ 0.001 were described as DEGs by the PoissonDis method^41^. KEGG functional analysis showed that DEGs were enriched for each term in the KEGG database, and the number of unigenes in each pathway was calculated. Pathways showing significant enrichment among the DEGs compared to the entire *A. argyi* transcriptome were identified using the hypergeometric test^42^. In this test, the p-value was calculated as follows:

$$p=1-\sum _{i=0}^{m-1}(M{\rm{i}})(N-Mn-i)/(Nn)$$

where M, N, m and n represent the number of annotated unigenes corresponding to each KEGG term, all unigenes with KEGG annotations, DEGs in M and DEGs in N, respectively.

### Transcription factor analysis

After detecting the ORF of each unigene with Getorf (parameter: -minsize 150)^43^, ORFs were aligned to TF protein domains in PlnTFDB (plant transcription factor database) using Hmmsearch with the default parameters^44^. The abilities of the unigenes to encode proteins were evaluated based on the characteristics of TF families described in PlnTFDB.

### Molecular cloning candidate genes for terpenoid biosynthesis

Seven unigenes (CL11002.Contig1, CL11002.Contig2, Unigene7594, Unigene11038, CL1159.Contig3, Unigene5791, CL1787.Contig4) encoding seven enzymes, ArHMGR1, ArHMGR2, ArMVD, ArDXS, ArDXR, ArHDS and ArHDR, respectively, were identified from the *A. argyi* transcriptomic data. The ORFs of these enzymes were amplified by PCR using gene-specific primers (Supplementary Table S5). The PCR conditions included an initial denaturation step at 94 °C for 2 min followed by 35 cycles of 98 °C for 30 s, annealing temperature (ArHMGR1/ArHMGR2, 55 °C; ArMVD, 52.5 °C; ArDXR, 53 °C; ArDXS/ArHDS/ArHDR, 57 °C) for 40 s and 68 °C for 1 min, and an additional extension of 68 °C for 7 min. The gene amplification efficiencies were determined by agarose gel electrophoresis, and the gene fragments were sub-cloned into the vector pMD19-T. The recombinant plasmids were confirmed by sequencing (Sangon Biotech, Shanghai, China).

### Accession code

The RNA-seq dataset for the three *A. argyi* tissues has been deposited into the NCBI Gene Expression Omnibus (GEO) database under accession code GSE102404.

## Electronic supplementary material

Supplementary information
