# Supplementary material for: De novo assembly and analysis of the Artemisia argyi transcriptome and identification of genes involved in terpenoid biosynthesis
Source: Sci Rep. 2018 Apr 11;8:5824. doi: 10.1038/s41598-018-24201-9 (PMC5895812; doi:10.1038/s41598-018-24201-9)
Supplement: Supplementary file 1 — Supplementary information [file 41598_2018_24201_MOESM1_ESM.pdf]

De novo assembly and analysis of the *Artemisia argyi* transcriptome and identification of genes involved in terpenoid biosynthesis

Miaomiao Liu[1,†], Jinhang Zhu[2,†], Shengbing Wu[3,4], Chenkai Wang[1], Xingyi Guo[5], Jiawen Wu[3,6,*]&Meiqi Zhou[3,4,*]

[1]Graduate School, Anhui University of Chinese Medicine, Hefei 230038, China

[2]Department of Physiology, School of Basic Medical Sciences, Anhui Medical University, Hefei 230032, China

[3]Key Laboratory of Xin'an Medicine, Ministry of Education, Anhui University of Chinese Medicine, Hefei 230038, China

[4]Institute of Acu-moxibustion and Meridian-collaterals, Anhui University of Chinese Medicine, Hefei 230038, China

[5]Division of Epidemiology, Department of Medicine, Vanderbilt University School of Medicine, Nashville, TN, 37232, USA

[6]Synergetic Innovation Center of Anhui Authentic Chinese Medicine Quality Improvement, Hefei 230038, China

*Address correspondence to Jiawen Wu, Ph.D.

Key Laboratory of Xin'an Medicine, Ministry of Education, Anhui University of Chinese Medicine, Hefei 230038, China.

Fax: +86-551- 65169371, Tel.: +86-551- 65169752.

Email: wujiawen@ahtcm.edu.cn

*Address correspondence to Meiqi Zhou, Ph.D.

Institute of Acu-moxibustion and Meridian-collaterals, Anhui University of Chinese Medicine, Hefei 230038, China.

Fax: +86-551-68129061, Tel.: +86-551-68129061.

Email: meiqizhou@163.com

[†]These authors contributed equally to this work.

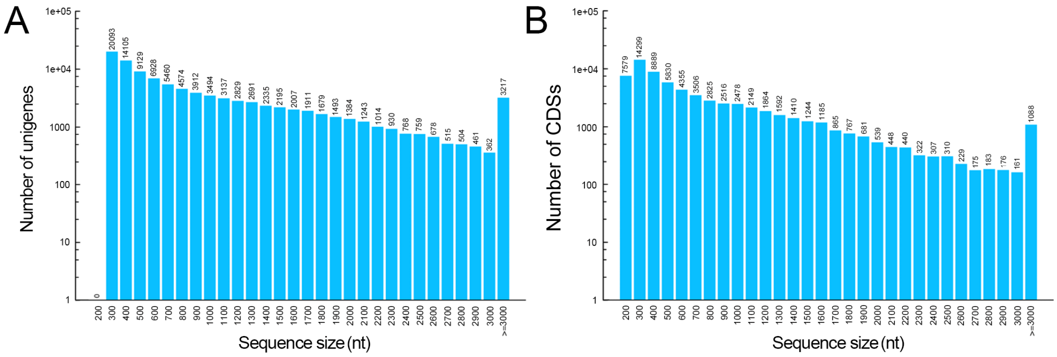

Supplementary Figure S1. Overview of assembly of the *A. argyi* transcriptome. (A) Size distribution of unigenes. (B) Size distribution of CDSs.

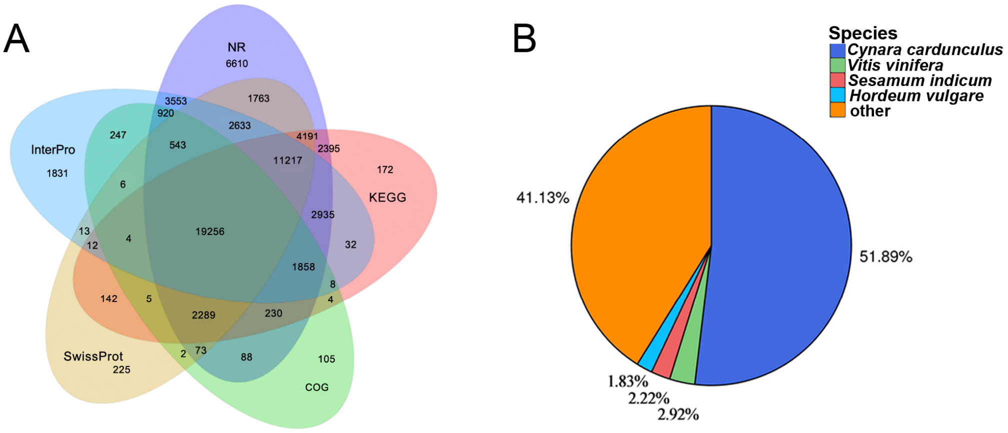

Supplementary Figure S2. (A) Venn diagram of annotated unigenes from the different databases. (B) Species distribution of *A. argyi* homologues against the Nr database.

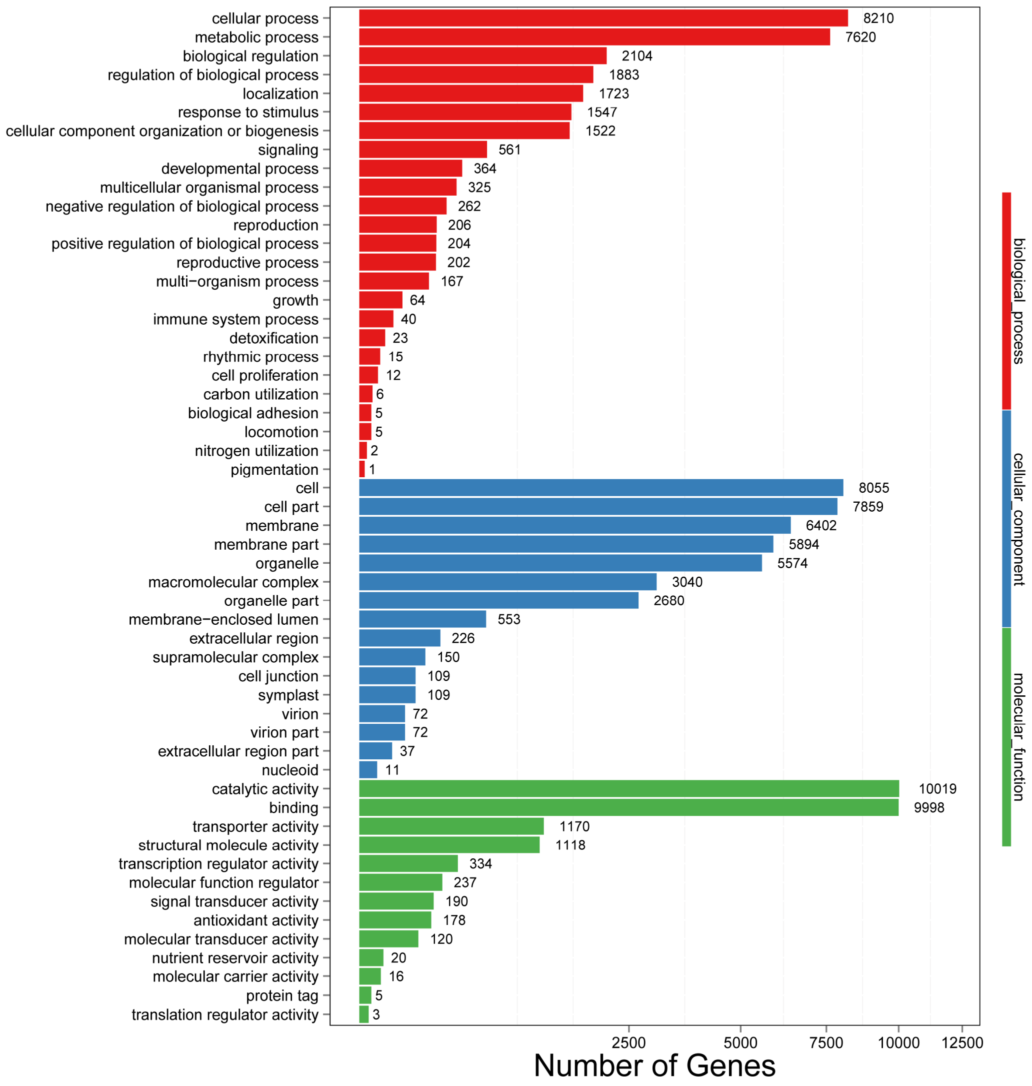

Supplementary Figure S3. GO functional classifications of *A. argyi* unigenes.

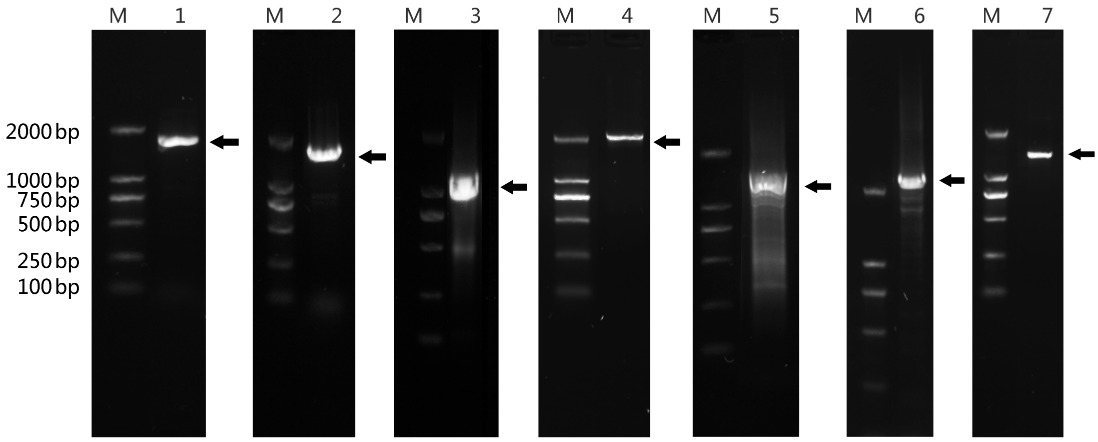

Supplementary Figure S4. Molecular cloning of seven genes. M represents the DNA marker, and 1, 2, 3, 4, 5, 6 and 7 represent ArHMGR1, ArHMGR2, ArMVD, ArDXS, ArDXR, ArHDS and ArHDR, respectively. The arrows indicate the target genes.

Supplementary Table S1. Quality evaluation of clean data.

Tissue type	Number of raw reads	Number of clean reads	Clean reads Q20 (%)	Clean reads ratio (%)
Leaf	86,560,026	74,033,662	98.63	85.53
Root	88,193,576	74,546,432	98.62	84.53
Stem	86,559,876	74,462,236	98.62	86.02

Supplementary Table 2. Qualitative unigene index.

Tissue type	Total number	Total length (bp)	Mean length (bp)	N50 (bp)	GC (%)
Leaf	59,626	58,446,995	980	1492	40.08
Root	60,599	47,722,360	787	1197	42.43
Stem	65,440	61,247,220	935	1405	39.95
AllUnigenes	99,807	92,726,525	929	1456	40.79

Supplementary Table S3. GOSlim functional analysis of 603 unigenes with leaf-specific expression.

Ontology	GO term	Number
Biological process	biosynthetic process	46
Biological process	cellular nitrogen compound metabolic process	38
Biological process	small molecule metabolic process	24
Biological process	single-organism biosynthetic process	23
Biological process	cellular protein modification process	19
Biological process	carbohydrate metabolic process	18
Biological process	response to stress	12
Biological process	single-organism carbohydrate metabolic process	11
Biological process	generation of precursor metabolites and energy	11
Biological process	lipid metabolic process	11
Biological process	translation	10
Biological process	cofactor metabolic process	9
Biological process	catabolic process	8
Biological process	single-organism catabolic process	7
Biological process	transport	7
Biological process	sulfur compound metabolic process	6
Biological process	protein folding	6
Biological process	biological_process	6
Biological process	homeostatic process	5
Biological process	signal transduction	5
Biological process	anatomical structure development	5
Biological process	photosynthesis	4
Biological process	single-organism transport	4
Biological process	cellular amino acid metabolic process	3
Biological process	tRNA metabolic process	3
Biological process	DNA metabolic process	3
Biological process	secondary metabolic process	3
Biological process	cell differentiation	2
Biological process	mRNA processing	2
Biological process	cell wall organization or biogenesis	2
Biological process	protein targeting	2
Biological process	cell division	2
Biological process	reproduction	2
Biological process	ribosome biogenesis	2
Biological process	cellular component assembly	1
Biological process	cell morphogenesis	1
Biological process	immune system process	1
Biological process	transmembrane transport	1
Biological process	nucleocytoplasmic transport	1

Biological process	protein complex assembly	1
Biological process	vesicle-mediated transport	1
Molecular function	ion binding	88
Molecular function	oxidoreductase activity	41
Molecular function	kinase activity	35
Molecular function	molecular_function	25
Molecular function	transmembrane transporter activity	21
Molecular function	substrate-specific transmembrane transporter activity	20
Molecular function	DNA binding	20
Molecular function	substrate-specific transporter activity	19
Molecular function	transferase activity, transferring glycosyl groups	12
Molecular function	transcription factor activity, sequence-specific DNA binding	11
Molecular function	RNA binding	10
Molecular function	lyase activity	8
Molecular function	structural constituent of ribosome	8
Molecular function	ATPase activity	6
Molecular function	peptidase activity	5
Molecular function	GTPase activity	5
Molecular function	methyltransferase activity	5
Molecular function	ligase activity	5
Molecular function	unfolded protein binding	4
Molecular function	phosphatase activity	4
Molecular function	nuclease activity	4
Molecular function	translation factor activity, RNA binding	4
Molecular function	transferase activity, transferring acyl groups	3
Molecular function	hydrolase activity, acting on glycosyl bonds	3
Molecular function	transcription factor activity, protein binding	3
Molecular function	enzyme regulator activity	3
Molecular function	isomerase activity	3
Molecular function	helicase activity	3
Molecular function	lipid binding	2
Molecular function	rRNA binding	2
Molecular function	transcription factor binding	1
Molecular function	transferase activity, transferring alkyl or aryl groups	1
Molecular function	nucleotidyltransferase activity	1
Molecular function	nucleic acid binding transcription factor activity	1
Molecular function	cytoskeletal protein binding	1
Molecular function	hydrolase activity, acting on carbon-nitrogen bonds	1
Molecular function	signal transducer activity	1
Cellular component	cellular_component	83
Cellular component	plastid	39
Cellular component	plasma membrane	22
Cellular component	nucleus	22

Cellular component	cytosol	15
Cellular component	cytoplasm	12
Cellular component	thylakoid	11
Cellular component	ribosome	9
Cellular component	protein complex	7
Cellular component	mitochondrion	6
Cellular component	intracellular	6
Cellular component	extracellular region	5
Cellular component	Golgi apparatus	5
Cellular component	endoplasmic reticulum	4
Cellular component	vacuole	3
Cellular component	cell	3
Cellular component	nucleolus	3
Cellular component	cell wall	2
Cellular component	cytoskeleton	1
Cellular component	organelle	1
Cellular component	endosome	1

Supplementary Table S4. KEGG annotation of leaf vs. root and leaf vs. stem DEGs.

Pathway	No. of DEGs (Leaf vs. Root)	No. of DEGs (Leaf vs. Stem)
Metabolic pathways	3657	2784
Biosynthesis of secondary metabolites	2182	1728
Plant-pathogen interaction	868	634
Endocytosis	756	529
RNA transport	741	425
Ribosome	688	440
Spliceosome	673	445
Protein processing in endoplasmic reticulum	632	409
Biosynthesis of amino acids	504	379
Carbon metabolism	497	360
Starch and sucrose metabolism	475	399
Plant hormone signal transduction	450	381
Phenylpropanoid biosynthesis	360	256
mRNA surveillance pathway	355	267
Purine metabolism	338	227
Glycolysis / Gluconeogenesis	305	226
RNA degradation	279	200
Pyrimidine metabolism	270	187
Amino sugar and nucleotide sugar metabolism	244	209
Ribosome biogenesis in eukaryotes	215	137
Oxidative phosphorylation	210	102
Cysteine and methionine metabolism	205	140
Glycerophospholipid metabolism	203	181
Ubiquitin mediated proteolysis	197	139
Pentose and glucuronate interconversions	195	143
Pyruvate metabolism	192	156
Circadian rhythm – plant	181	141
Aminoacyl-tRNA biosynthesis	180	131
Fatty acid metabolism	177	120
Glyoxylate and dicarboxylate metabolism	175	111
Carbon fixation in photosynthetic organisms	164	128
Peroxisome	162	129
Glycerolipid metabolism	157	115
Flavonoid biosynthesis	152	140
Glycine, serine and threonine metabolism	151	130
DNA replication	151	139
Nucleotide excision repair	147	142
ABC transporters	146	118
RNA polymerase	144	94

Stilbenoid, diarylheptanoid and gingerol biosynthesis	143	135
Galactose metabolism	142	114
Homologous recombination	139	125
Phagosome	136	90
Ascorbate and aldarate metabolism	130	103
Glutathione metabolism	129	84
Mismatch repair	129	115
Pentose phosphate pathway	127	79
Fructose and mannose metabolism	121	95
alpha-Linolenic acid metabolism	121	99
2-Oxocarboxylic acid metabolism	117	87
Cyanoamino acid metabolism	117	121
Valine, leucine and isoleucine degradation	115	76
Tyrosine metabolism	114	93
Nitrogen metabolism	111	78
Regulation of autophagy	110	79
Fatty acid degradation	109	75
Biosynthesis of unsaturated fatty acids	109	66
Porphyrin and chlorophyll metabolism	108	94
Limonene and pinene degradation	108	96
Citrate cycle (TCA cycle)	106	62
Alanine, aspartate and glutamate metabolism	105	74
Phosphatidylinositol signaling system	105	98
beta-Alanine metabolism	99	75
Carotenoid biosynthesis	97	811
Base excision repair	93	60
Phenylalanine metabolism	89	72
Arginine biosynthesis	85	57
Inositol phosphate metabolism	84	76
Arginine and proline metabolism	83	64
Terpenoid backbone biosynthesis	82	78
Other glycan degradation	82	92
Fatty acid biosynthesis	82	62
Tryptophan metabolism	81	70
Photosynthesis	78	64
Ubiquinone and other terpenoid-quinone biosynthesis	77	50
Sphingolipid metabolism	73	65
N-Glycan biosynthesis	71	47
Tropane, piperidine and pyridine alkaloid biosynthesis	69	48
Phenylalanine, tyrosine and tryptophan biosynthesis	69	59

Isoquinoline alkaloid biosynthesis	68	52
Propanoate metabolism	66	43
Basal transcription factors	66	47
Protein export	65	43
Ether lipid metabolism	65	61
Proteasome	65	51
Flavone and flavonol biosynthesis	63	68
Sulfur metabolism	63	59
Zeatin biosynthesis	62	60
Lysine degradation	59	39
Arachidonic acid metabolism	57	32
Lysine biosynthesis	55	48
Cutin, suberine and wax biosynthesis	52	59
Nicotinate and nicotinamide metabolism	50	27
Diterpenoid biosynthesis	50	47
Pantothenate and CoA biosynthesis	48	31
Selenocompound metabolism	47	23
Butanoate metabolism	47	40
Isoflavonoid biosynthesis	43	44
Steroid biosynthesis	43	31
Linoleic acid metabolism	40	32
Biotin metabolism	39	31
Histidine metabolism	38	27
Valine, leucine and isoleucine biosynthesis	37	28
Fatty acid elongation	34	25
Vitamin B6 metabolism	31	21
One carbon pool by folate	31	20
SNARE interactions in vesicular transport	28	27
Degradation of aromatic compounds	27	19
Monoterpenoid biosynthesis	27	27
Sesquiterpenoid and triterpenoid biosynthesis	27	45
Photosynthesis – antenna proteins	26	12
Glycosphingolipid biosynthesis–ganglio series	26	33
Glycosaminoglycan degradation	25	40
Riboflavin metabolism	22	13
Brassinosteroid biosynthesis	21	13
C5-Branched dibasic acid metabolism	20	17
Benzoxazinoid biosynthesis	18	18
Glycosylphosphatidylinositol(GPI)-anchor biosynthesis	17	11
Folate biosynthesis	16	11
Taurine and hypotaurine metabolism	15	12
Non-homologous end-joining	15	10
Anthocyanin biosynthesis	15	20

Synthesis and degradation of ketone bodies	14	17
Other types of O-glycan biosynthesis	14	20
Thiamine metabolism	13	10
Lipoic acid metabolism	13	13
Indole alkaloid biosynthesis	12	10
Sulfur relay system	11	10
Glycosphingolipid biosynthesis–globo series	8	5
Betalain biosynthesis	4	3
Caffeine metabolism	1	0

Supplementary Table S5. Gene-specific primers for key enzymes involved in terpenoid biosynthesis.

Gene name	Forward primer (5'-3')	Reverse primer (5'-3')
ArHMGA1	ATGGATGTTCGCCGGCGTCCTGTTAAT	CTAGGAAGAGCTTAACTTGGTCATATCTCTAGTGG
ArHMGA2	ATGGATGTCCGAAGACGATCTTCGTTGAAACC	TTAAGCCTTGGAAGCAAGATCTCTGGTGGAGC
ArMVD	ATGGGAAGTGAGTTAGAAAAATGGGTGTTAAT	TTACTTCGGCAAGCCAGTGAGTGGGTTGAGGA
ArDXS	ATGGCTTCCTGTGGTGCTTTGAAGGGTG	CTATGCATTGACAGCTTCTAGCAGTCTTTCCTTAC
ArDXR	ATGTCTTTGAACACCCTTTCCCCTTCAG	TCATACTAGTGCCGGAGTTAAACCAGATGAT
ArHDS	ATGGCGACCGGGGCTGTTCCAGCT	CTACTCTTCAACTGGAGGATCGACCCAGC
ArHDR	ATGGCGTCTTTGCAGCTCACACCTCTTTC	CTACACCAATTGCAGGGCTTCCTCACG
